# Os Vesalinum Mistaken for Fracture of Fifth Metatarsal

**Published:** 1914-08

**Authors:** 


**Os Vesalinum Mistaken for Fracture of Fifth Metatarsal.** Wm. Pearce Coues, Boston M. & S. Jour., May 7, 1914, describes this rare anomaly as shown by X-rays, and refers to the literature.

